# What Is Valued Most by Patients With Type 2 Diabetes Mellitus When Selecting Second-Line Antihyperglycemic Medications in China

**DOI:** 10.3389/fphar.2021.802897

**Published:** 2021-12-23

**Authors:** Shimeng Liu, Jing Liu, Yijiang Yu, Lei Si, Chengxiang Tang, Zhigang Liu, Yingyao Chen

**Affiliations:** ^1^ School of Public Health, Fudan University, Shanghai, China; ^2^ NHC Key Laboratory of Health Technology Assessment (Fudan University), Shanghai, China; ^3^ School of Management, Hainan Medical University, Haikou, China; ^4^ Huai’an Hospital of Traditional Chinese Medicine, Huai'an, China; ^5^ The George Institute for Global Health, UNSW Sydney, Kensington, NSW, Australia; ^6^ School of Public Administration, Guangzhou University, Guangzhou, China

**Keywords:** best-worst scaling, patient preferences, type 2 diabetes mellitus, second-line antihyperglycemic medications, China

## Abstract

**Objective:** To estimate patient preferences for second-line antihyperglycemic medications in China.

**Methods:** A face to face survey with the best-worst scaling (BWS) choices was administered in patients with diagnosed type 2 diabetes mellitus (T2DM). Study participants were asked to indicate which attribute they valued most and which attribute they valued least in 11 choice sets, each of which consisted of five alternatives out of 11 antihyperglycemic medication-specific attributes (treatment efficacy, weight change, hypoglycemic events, gastrointestinal side effects, cardiovascular health, urinary tract infection and genital infection side effects, edema, mode of administration, bone fracture, dosing frequency and out-of-pocket cost). A counting approach, a conditional logit model, and K-means clustering were used to estimate the relative importance of items and preference heterogeneity.

**Results:** A total of 362 participants were included with a mean age of 63.6 (standard deviation: 11.8) years. There were 56.4% of participants were women, and 56.3% being diagnosed with diabetes for at least 5 years. Efficacy, cardiovascular health and hypoglycemic events were valued most, while dosing frequency, mode of administration and bone fracture were valued least. The K-means clustering further showed preference heterogeneity in out-of-pocket cost across the participants.

**Conclusion:** Our study suggests that treatment efficacy, cardiovascular health and hypoglycemic events are valued most by Chinese patients with T2DM when selecting second-line antihyperglycemic medications. The study improves the understanding of patients’ preferences for second-line antihyperglycemic medications in China.

## Introduction

Diabetes is a chronic health issue in China, with the prevalence increased from 9.7% in 2007 to 11.2% in 2017 among Chinese adults (Li et al., 2020). Patients with diabetes in China were found with a low health literacy, which likely leads to poorer health outcomes and poorer use of health care services (Wei et al., 2020). Another barrier to patient’s access to diabetes care was that patients needed to pay out-of-pocket for some of the in-hospital services or antihyperglycemic medications and many patient families suffered from catastrophic health expenditure (Wu et al., 2019). Several healthcare reforms were conducted in 2013 to address these issues. For example, prevention and treatment of diabetes in China has moved from tertiary hospitals to community health service centres, from simple clinical treatment to tertiary prevention of diabetes, and from simple control of blood glucose to control of weight, blood glucose, blood pressure, and blood lipids. (Li et al., 2020).

Among all types of diabetes, type 2 diabetes mellitus (T2DM) is a complex, chronic illness requiring continuous medical care with multifactorial risk-reduction strategies beyond glycemic control. Established guidelines recommend that once initiated, metformin should be continued as long as it is tolerated and not contraindicated (Doyle-Delgado et al., 2020). However, given the progressive nature of the disease, within 3 years of receiving monotherapy, 50% of patients are inadequately controlled and require add-on therapies (Turner et al., 1999). For patients without established atherosclerotic cardiovascular disease (ASCVD), indicators of high ASCVD risk, heart failure, or chronic kidney disease, the choice of a second agent to add to metformin is not yet guided by empirical evidence (Doyle-Delgado et al., 2020). Drug choice should be personalized based on personal clinical condition and their preferences. For example, if the main driver of the treatment choice is out-of-pocket cost, a sulfonylurea or thiazolidinedione (TZD) might be considered as they are currently reimbursed in China; if the main concern is to avoid hypoglycemia, glucagon-like peptide-1 receptor agonist (GLP-1 RAs), sodium-glucose cotransporter two inhibitor (SGLT2i), dipeptidyl peptidase Ⅳ inhibitor (DPP-4i), or a thiazolidinediones (TZD) might be preferred (Doyle-Delgado et al., 2020).

The 2021 American Diabetes Association guidelines highlighted the importance of taking patient preferences into prescription decision making. Balancing medical and personal needs is a key aspect of shared decision making. This is a process involving two-way information giving (medical and personal) between the clinician and the patient concerning all available options (Jordan et al., 2002). An individual patient may place different levels of importance on the various aspects of drug therapy. For instance, some may emphasize the benefits of glycemic control, whereas others may place more emphasis on the risk of hypoglycemic events. Given the choices that are available to patients, trade-offs should be made based on various risks, benefits and convenience factors. In clinical practice, treatment decisions are often not based on what the patient wishes, examples from diabetes management exist in which patient preference towards, and healthcare providers judgment of, a successful treatment do not coincide (Porzsolt et al., 2010). A priori understanding of patient preferences has become vitally important because incorporating patient preferences helps to capture a perspective that cannot be gathered through clinical trial data which will impact treatment adherence and patient satisfaction (Egbrink and M, 2014).

Patient preferences can be quantitatively determined through choice modelling techniques, such as best-worst scaling (BWS) or discrete choice experiment (DCE). There have been several DCEs conducted in patients with diabetes (Purnell et al., 2014; von Arx and Kjeer, 2014); however, BWS studies in this field are rare (Cheung et al., 2016; Crossnohere et al., 2021). BWS is rooted in random utility theory, a well-tested theory of human decision making hypothesised by Thurstone (Thurstone, 1927) and generalised by McFadden (McFadden, 1974). BWS can be used to determine preferences for a wide range of health care questions, by asking the respondent to indicate the best and the worst in a set of available items or options. It has been shown that BWS estimates the utilities of all but one of the attribute levels in a best–worst choice experiment. This enables the impact of all but one attribute to be estimated, where impact of an attribute is the average across all its levels, which traditional ‘pick one’ DCEs cannot do (Flynn et al., 2007). There are three types of BWS, which differ in terms of the complexity of the items or options under consideration: BWS object case (case 1), BWS profile case (case 2) and BWS multi-profile case (case 3). BWS object case can be very attractive in health care because health care goods/services can be complicated, and even pairs of specifications (e.g., a simple DCE) may lead to an unacceptable cognitive burden, particularly among vulnerable patient groups (Flynn, 2010).

Crossnohere et al. (Crossnohere et al., 2021) have compared the preferences of patients and the general public for treatment outcomes of T2DMs using BWS in United States. However, only seven attributes were included in this study which may not fully capture the profile of various antihyperglycemic drugs for T2DM (e.g., gastrointestinal side effects). In addition, one additional important takeaway message from these previous studies is that preferences can vary between cultures, and if patient preferences are to be considered in prescribing decisions, they need to be specific to the cultural context (Donnan et al., 2020). The understanding of patient preferences for second-line antihyperglycemic medicines is still lacking in China now. In this study, we aimed to close the research gap by investigating various factors that affect medication preferences when selecting a second-line antihyperglycemic medicine in Chinese patients with T2DM.

## Materials and Methods

### Study Participants and Elicitation Method

A face-to-face survey was undertaken in Huaian in Jiangsu province and Danzhou in Hainan province from March to June in 2021. In each city, two hospitals and two primary care institutions were selected according to the convenience sampling. Finally, one tertiary hospital and one secondary hospitals from urban areas, two primary care institutions from village or rural areas were selected in each province. Participants were eligible to complete the survey if they were Chinese, ≥18 years of age and diagnosed with T2DM by a health-care professional. Individuals were asked to participate regardless of their medication history.

There is no consensus on the minimal sample size for a BWS (Flynn et al., 2007; Cheung et al., 2016). In our study, we aimed to recruit 100 participants as the minimal sample size (Pearmin et al., 1991) which has also been used in other BWS studies (Flynn, 2010). To ensure the quality of data collection, we collect the data *via* a face-to-face manner. In the tertiary hospital, the questionnaire was administered to patients by the clinicians and the research team, while in the primary care institution the face-to-face survey was conducted in a conference room or a waiting room by the research team. The instructions of the BWS and the rationale of the survey were also explained in detail by one or two investigators who received specific training by the research team. For those respondents who had difficulty in filling in the questionnaire—for example, the patients who suffered from poor eyesight—respondents were offered the opportunity to complete the questionnaire verbally. A pilot study was conducted with six T2DM patients (the research team were familiar with) before data collection to examine the comprehensibility, acceptability, and validity of the questionnaire with language and the layout revised thereafter. The patients completed the questionnaire themselves anonymously after they provided informed written consent. The completion of the questionnaire took about 15–20 min and all completed questionnaires were returned directly to the investigators.

The ethics approval was obtained from the ethics review board of the School of Public Health at Fudan University (Reference No. IRB# 2021-07-0911), and the research adhered to the tenets of the Declaration of Helsinki. All patients gave their informed consent prior to their inclusion in the study.

### Selection of Antihyperglycemic Medication-Specific Factors

A range of medications in the form of oral and/or injectable are available to control blood glucose (Tan et al., 2019). This variety of treatment options naturally provide a diversity of clinical efficacy, modes of administration, and adverse event profiles. One of the principles of determining attributes in our study was to include as many profiles as the antihyperglycemic drugs. A search of the medical literature was conducted using PubMed, Web of Science and CNKI (Chinese database) to identify any stated preference studies that had been previously published in T2DM (Gelhorn et al., 2013; Purnell et al., 2014; von Arx and Kjeer, 2014; Donnan et al., 2020; Ozdemir et al., 2020; Igarashi et al., 2021) and the guidelines (Jia et al., 2019; Doyle-Delgado et al., 2020; Society, 2021) for the prevention and control of T2DM. This literature review helped to generate an initial list of attributes. To further refine the attributes list and to identify possible gaps, we have conducted focus group discussions with clinicians and reviewed the package inserts of antihyperglycemic medicines that were available to the Chinese patients at the time. During the focus group discussion, clinicians were asked to discuss the initial list of nine attributes that were developed based on the literature review. They were also asked to provide additional attributes for potential inclusion in the final attributes list. After the discussion, the mode of administration and cardiovascular health were further added as two new attributes, and the urinary tract infection was adjusted to urinary tract infection and genital infection side effects. Finally, 11 antihyperglycemic medication-specific factors were determined, including treatment efficacy on HbA1c, hypoglycemic events, mode of administration, dosing frequency, out-of-pocket cost, and side effects such as gastrointestinal side effects, cardiovascular health, urinary tract infection (UTI) and genital infection side effects, weight change, edema and bone fracture. The final set of attributes and the explanation are provided in Table 1.

**TABLE 1:** Attributes in the BWS: antihyperglycemic medication-specific factors.

	Attributes	Description
1	Treatment efficacy	Different diabetes drugs have different efficacies for reducing the HbA1c; for example, insulin has the highest efficacy, GLP-1 RAs, TZD and sulfonylureas have high efficacy, SGLT2i and DPP-4i has an intermediate efficacy
2	Weight change	Some diabetes drugs can reduce weight (e.g., SGLT2i, GLP-1 RAs), while some can increase weight (e.g., TZD, sulfonylureas, insulin)
3	Hypoglycemic events	Hypoglycemic events happen when blood sugar level goes too low. Symptoms include tiredness, dizziness, confusion, increased heart rate, and a cold, clammy feeling. Sulfonylureas, glinides and insulin can increase the risks of hypoglycemic events
4	Gastrointestinal side effects	Some diabetes drugs such as glucosidase inhibitor, GLP-1 RAs may cause gastrointestinal side effects (e.g., nausea, vomit, diarrhea)
5	Cardiovascular health	Medication-related change in cardiovascular health (e.g. risk of heart attack or stroke). Some diabetes drugs, for example, GLP-1 RAs may have cardiovascular benefits
6	UTI and genital infection side effects	Some diabetes drugs (e.g., SGLT2i) may cause UTI or genital infection during medication
7	Edema	Some diabetes drugs such as TZD are associated with an increased risk of fluid retention (edema)
8	Mode of administration	Pill or injectable
9	Dosing frequency	The number of times that patient needs to take diabetes drugs within a certain time
10	Bone fracture	Some diabetes drugs, for example, the use of TZD or SGLT2i is associated with an increased risk of bone fracture
11	Out-of-pocket cost	The cost of diabetes drugs that patients need to pay out-of-pocket.

Abbreviations: BWS, best-worst scaling; GLP-1 RAs, glucagon-like peptide-1, RAs, receptor agonist; TZD, thiazolidinediones; SGLT2i, sodium-glucose cotransporter two inhibitor; DPP-4i, dipeptidyl peptidase IV inhibitor; UTI, urinary tract infection.

### Experimental Design

BWS is one research method to measure ratings involving trade-offs among many items. This method is also known as “MaxDiff” (Szeinbach et al., 1999). BWS researchers recommend structuring these series of blocks in balanced incomplete block designs (BIBD) (Louviere et al., 2013). So, in our study, the BWS experiment design was developed using a BIBD in R, which is a type of design in which a subset of treatments is assigned to each block. Each patient was required to select in each of 11 choice sets. Each choice set consisted of five alternatives out of 11 antihyperglycemic medication-specific factors. The complete design matrix is provided in the Appendix. Additional questions including patient socio-demographics (e.g., age, sex, marital status, usual place of residence, annual family income, education level), duration of diabetes, and current medication use were also collected.

### Statistical Analysis

Two approaches were used in analyzing the BWS data: a counting approach and a modelling approach. The counting approach calculates several types of scores based on the number of times (the frequency or count) that item *i* (the 11 antihyperglycemic drug -specific factors) is selected as the best and the worst in all the questions for respondent *n*. Such scores are roughly divided into two categories: disaggregated (individual-level) scores and aggregated (total-level) scores. A previous study showed that the B-W score was a good approximation of the precision of the maximum likelihood estimate in the logit model (Marley and Louviere, 2005). In addition to the counting analysis, we also used a modelling approach using the McFadden’s conditional logistic regression (CL) to analyze the responses. We compared the CL and counting estimates to examine the correlation between the two approaches. Finally, we divided the respondents into two groups to investigate the preference heterogeneity across our sample by using the KMeans clustering. The KMeans clustering method is a traditional cluster approach that involves minimizing within-cluster variance and maximizing across cluster variance.

## Results

### Characteristics of Study Participants

A total of 423 participants were recruited in our study, 406 (96.0%) consented and agreed to participate in the survey (the reason for the non-participates was complicated, e.g. distrust in health practitioners, time constraints, inadequate interest in this survey, etc.). Finally, 362 (89.2%) participants from four hospitals (*n* = 177) and four primary care institutions (*n* = 185) completed the questionnaire and were included in the analysis. The demographic and clinical characteristics of the participants are presented in Table 2. The mean age was 63.6 (SD = 11.8) years. Most of the study participants were female (56.4%), were married (85.6%), were from rural areas (54.4%), were diagnosed with diabetes (56.3%) for at least 5 years and had at least two medications for diabetes (69.9%).

**TABLE 2:** Self-reported sociodemographic and clinical characteristics.

*n* = 362	*N*	%
Age (years), mean (SD) [range]	63.6( ± 11.8)[27–92]	—
Gender		
Male	158	43.7
Female	204	56.4
Place of origin		
Rural	197	54.4
Urban	165	45.6
Marital status		
Married	310	85.6
Single	9	2.5
Divorced	4	1.1
Widowed	39	10.8
Education level		
Primary school or below	165	45.6
Junior high school	99	27.4
Senior high school or above	98	27.0
Annual family income (CNY)		
Less than 20,000	159	43.9
20,000 to 40,000	98	27.1
50,000 to 70000	61	16.9
80,000 to 100,000	19	5.3
More than 100,000	25	6.8
BMI, mean (SD) [range]	24.0 (±3.4) [15.6–33.4]	
Number of medications currently taking for diabetes		
One medication	109	30.1
Two medications	159	43.9
Three medications	59	16.3
Four or more medications	35	9.7
Time since diagnosis of diabetes		
Less than 1 year	50	13.8
More than 1 year and up to 5 years	108	29.8
More than 5 years and up to 10 years	146	40.3
More than 10 years	58	16.0

Abbreviations: SD, standard deviation; CNY, chinese yuan.

### Results of BWS

**FIGURE 1:**
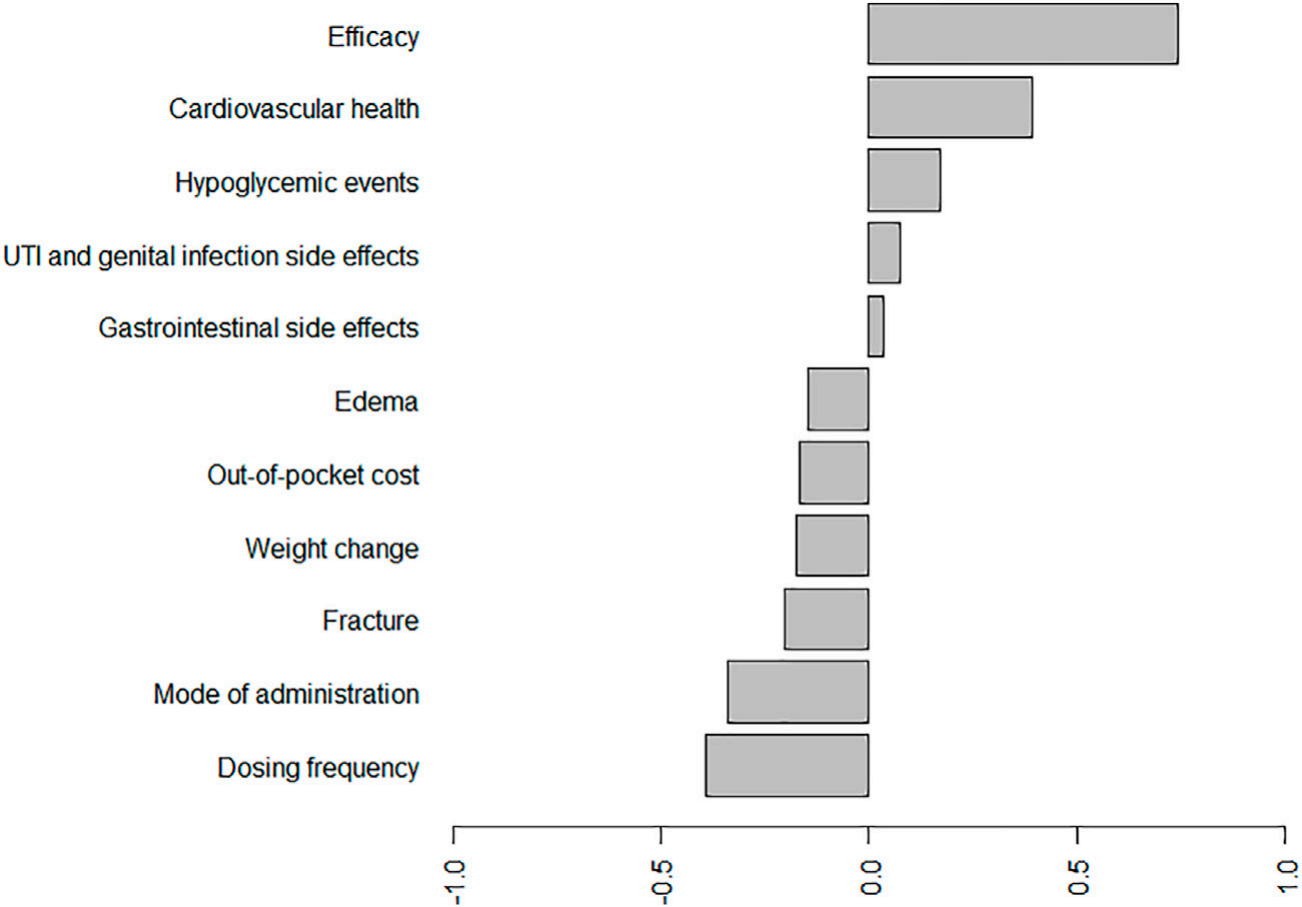
Standard BW scores for the antihyperglycemic medication-specific factors. Abbreviations: UTI, urinary tract infection.*On average, efficacy, cardiovascular health, hypoglycemic events, UTI and genital infection side effects, and gastrointestinal side effects have positive standardized BW scores, meaning that these roles were more frequently selected as the most important than the least important. According to these scores, the most important attribute in the patient’ medication preferences is, on average, efficacy, the second-most important is cardiovascular health, and the least important is dosing frequency.

The results of BWS by counting and CLM approaches are shown in Table 3. The top three important attributes evaluated by the average BW score were efficacy, cardiovascular health, and hypoglycemic events. In contrast, dosing frequency, mode of administration, and fracture were the three least important attributes. In addition, the mean standardized BW scores are plotted in Figure 1 to compare the relative importance of the included antihyperglycemic medication-specific attributes. Similar to the results in Table 3, the most important attribute in the patient’ medication preferences was efficacy. In total, six antihyperglycemic medication-specific factors have negative standardized BW scores (i.e., out-of-pocket cost, mode of administration, dosing frequency and side effects such as edema, weight change, bone fracture), meaning that these attributes were more frequently selected as the least important than the most important.

**TABLE 3:** Results of counting and CLM.

Attributes	Total counts				Individual proportion				—	CLM	
	Most important	Least important	BW scores	Standardized BW	Most important	Least important	BW scores	Standardized BW	SD	Coefficient	Rank
Efficacy	1356	14	1342	0.741	3.746	0.039	3.707	0.741	0.290	2.851***	1
Cardiovascular health	769	57	712	0.393	2.124	0.157	1.967	0.393	0.332	1.743***	2
Hypoglycemic events	439	131	308	0.170	1.213	0.362	0.851	0.170	0.350	1.031***	3
Gastrointestinal side effects	243	177	66	0.036	0.671	0.489	0.182	0.036	0.283	0.729***	4
UTI and genital infection side effects	290	152	138	0.076	0.801	0.420	0.381	0.076	0.280	0.716***	5
Edema	106	368	−262	−0.145	0.293	1.017	−0.724	−0.145	0.310	0.092	6
Out-of-pocket cost	328	625	−297	−0.164	0.906	1.727	−0.820	−0.164	0.580	0.000 (ref.)	7
Weight change	184	501	−317	−0.175	0.508	1.384	−0.876	−0.175	0.412	−0.003	8
Bone fracture	86	453	−367	−0.203	0.238	1.251	−1.014	−0.203	0.322	−0.111*	9
Mode of administration	96	708	−612	−0.338	0.265	1.956	−1.691	−0.338	0.336	−0.478***	10
Dosing frequency	85	796	−711	−0.393	0.235	2.199	−1.964	−0.393	0.351	−0.629***	11

***p < 0.01, **p < 0.05, *p < 0.1. Abbreviations: UTI, urinary tract infection; BW, best-worst; SD, standard deviation; CLM, conditional logit model.

**FIGURE 2:**
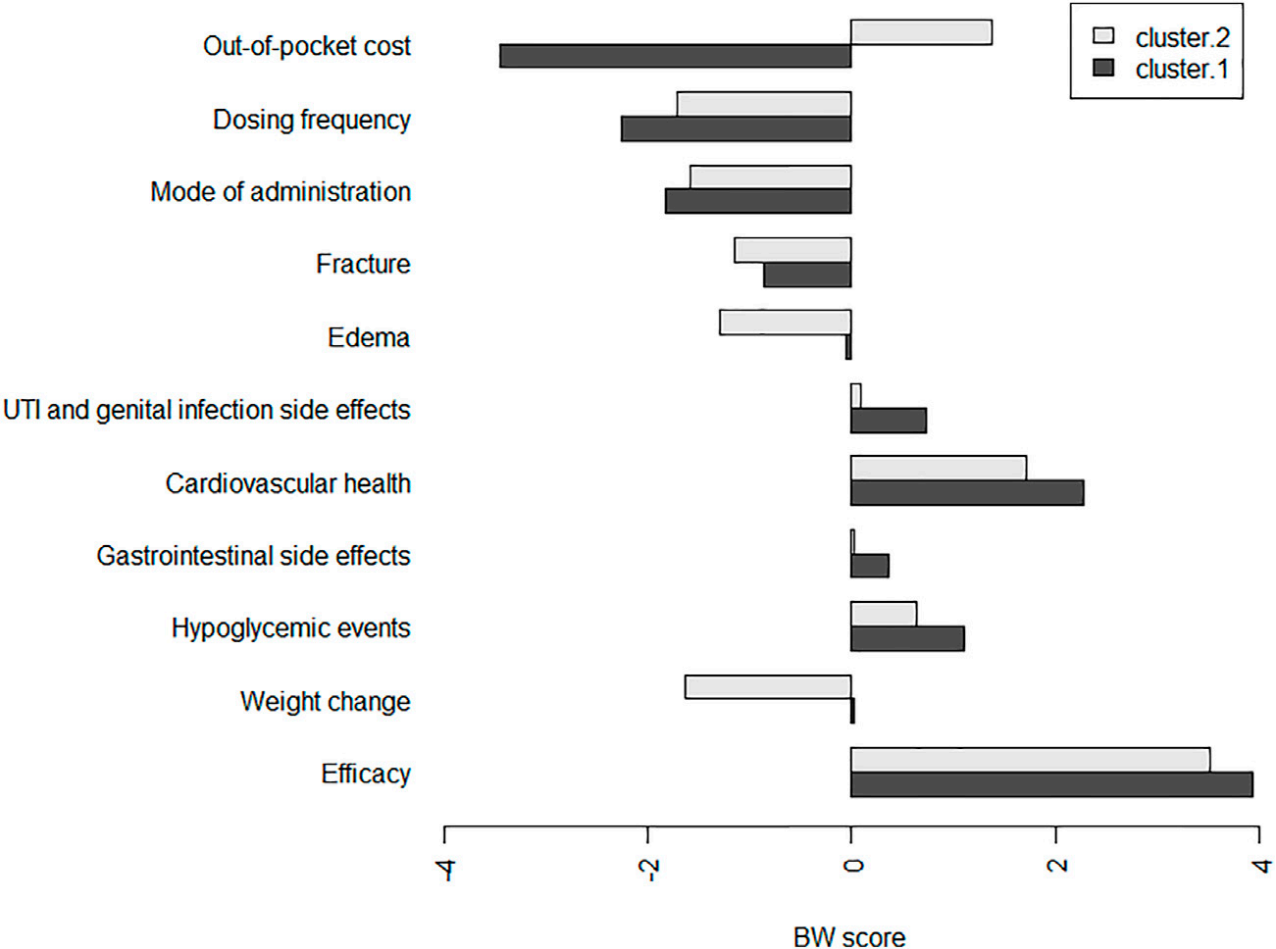
Individual BW scores per antihyperglycemic medication-specific factors in two clusters. Abbreviations: UTI, urinary tract infection.*The participants were classified into two groups using k-means clustering, which divides patients into k clusters (groups) according to their within group sum of squares. We set the disaggregated BW scores (BW) as the data for clustering and the number of clusters to two.

The coefficients of CLM were consistent with the results in the counting analysis. Efficacy was found as the important attribute for patients when selecting a second-line antihyperglycemic drug. Preferences for gastrointestinal side effects and UTI and genital infection side effects were not much different. The concordance between the results from the CLM and counting analysis was high (Supplementary Figure S2). The results of KMeans cluster in Figure 2 indicated relatively small differences in the BW scores between the two groups in terms of efficacy, cardiovascular health, mode of administration and dosing frequency. However, the difference in out-of-pocket cost attribute was found between the two clusters. The subgroup results further suggested that out-of-pocket cost led to a higher utility value for T2DM patients who were women, from a rural area and more than 60 years old (Supplementary Table S2).

## Discussion

To our knowledge, this is the first study to investigate treatment preferences of T2DM patients when selecting a second-line antihyperglycemic drug in China. Overall, patients value efficacy, cardiovascular health and hypoglycemic events most, while value dosing frequency and mode administration least when choosing a second-line antihyperglycemic medicine.

We have found that treatment efficacy, cardiovascular health and hypoglycemic events were the three most important attributes influencing T2DM patient preferences when selecting second-line antihyperglycemic drugs in China. The findings are consistent with another BWS study in the American population (Crossnohere et al., 2021), where the outcome HbA1c, cardiovascular health and hypoglycemic events were ranked as the most important attributes. In our study, patients demonstrated the strongest preference for treatment efficacy, and there were no major differences in importance ratings between the two clusters. Clinical evidence suggests that all available diabetes treatments today enable glucose control (Bennett et al., 2011; Maloney et al., 2019). Thus, it is likely that the strength of preference towards being in efficacy reflects the challenges patients face in achieving glucose control in daily life. These include issues such as maintaining treatment adherence and sustaining lifestyle changes (von Arx and Kjeer, 2014). When optimal glucose levels are achieved, the risk of long-term complications related to micro-and macrovascular damage is expected to decrease. Conversely, the risk of short-term complications, such as periods with low glucose levels (hypoglycemia), is expected increased (Davis and Alonso, 2004; Finfer et al., 2009). Thus, in clinical practice, healthcare providers are encouraged to balance patient preferences and health condition to achieve an optimal glucose control.

Numerous studies have shown the efficacy of controlling individual cardiovascular health factors in preventing or slowing ASCVD in people with diabetes. Although the use of antihyperglycemic medications are generally not associated with an increased risk, newer medication classes have demonstrated significant reduction in the composite endpoint of cardiovascular death, nonfatal myocardial infarction or nonfatal stroke, which made SGLT2i more popular among some T2DM patients (Zinman et al., 2015).

Dosing frequency and mode of administration carrying the least weight for patient treatment preferences. Our results were consistent with the review conducted by von et al. (von Arx and Kjeer, 2014), although fear of injections is common among non-insulin users, patients are not willing to trade efficacy to avoid injections. With the popularization of diabetes knowledge and the improvement of injection devices, more and more patients begin to realize that although oral medications may be more convenient, injectable agents often are needed to achieve treatment goals (Doyle-Delgado et al., 2020). In fact, most of the medicines with high treatment efficacy such as insulin and GLP-1 RAs are injectable.

Although out-of-pocket cost is only of a moderate preference, a relatively large heterogeneity in patient medication preferences was found between the two clusters. The cost of diabetes drugs has increased substantially during the past two decades (Riddle and Herman, 2018) which might put a hurdle to medicine adherence in patients with financial hardship (Kang et al., 2018). In China, the out-of-pocket cost for diabetes care is determined by the patient’s health insurance. There are two main public health insurances including the urban resident basic medical insurance for urban and rural residents, and urban employee basic medical insurance for urban employees. The benefit package and reimbursement level also vary across different regions. It suggested that the government should take some steps to increase the financial protection to these patients who are women, from rural area and more than 60 years old, for example, having more high costs medicines covered by public health insurance.

This study has two limitations to note. First, we did not include BWS quality control options, and all the complete questionnaires were included for the final analysis. Unlike DCEs, there is no distinct “good” or “bad” between the attributes within a choice set, so it was hard to set the dominant questions. To ensure the quality of data collection, we collect the data *via* a face to face mode. And nearly all of the attributes were statistically significant in influencing the treatment preferences of T2DM patients. Second, this study took place among patients in Hainan and Jiangsu provinces using a convenience sampling method for the data collection Our study participants might not be representative of all Chinese patients with T2DM, therefore our results might not be generalizable to other T2DM populations.

## Conclusion

Our study has found that efficacy, cardiovascular health and hypoglycemic events are the important attributes in T2DM patients when selecting second-line antihyperglycemic medicines in China. There is notable heterogeneity in patients’ medication preferences for the out-of-pocket cost attribute, suggesting that cost-reducing strategies could improve adherence in some cases. The results of this study will be useful for a better understanding of Chinse T2DM patients’ preferences when selecting second-line antihyperglycemic drugs, and to improve treatment adherence and patient satisfaction.

## Data Availability

The raw data supporting the conclusion of this article will be made available by the authors, without undue reservation.
