# Supplementary material for: What Is Valued Most by Patients With Type 2 Diabetes Mellitus When Selecting Second-Line Antihyperglycemic Medications in China
Source: Front Pharmacol. 2021 Dec 23;12:802897. doi: 10.3389/fphar.2021.802897 (PMC8733399; doi:10.3389/fphar.2021.802897)
Supplement: Supplementary file 1 [file DataSheet1.zip › Supplemental materials/802897_SupMaterial.DOCX]

**TABLE S1.** Balanced Incomplete Block Design (BIBD) matrix

| Choice set | Alt1 | Alt2 | Alt3 | Alt4 | Alt5 |
| --- | --- | --- | --- | --- | --- |
| No 1 | 2 | 5 | 7 | 9 | 10 |
| No 2 | 1 | 2 | 5 | 6 | 11 |
| No 3 | 1 | 2 | 4 | 8 | 10 |
| No 4 | 3 | 4 | 5 | 10 | 11 |
| No 5 | 1 | 3 | 5 | 8 | 9 |
| No 6 | 2 | 3 | 4 | 6 | 9 |
| No 7 | 1 | 4 | 7 | 9 | 11 |
| No 8 | 2 | 3 | 7 | 8 | 11 |
| No 9 | 1 | 3 | 6 | 7 | 10 |
| No 10 | 6 | 8 | 9 | 10 | 11 |
| No 11 | 4 | 5 | 6 | 7 | 8 |

*The first column indicates the numerical order of the choice set; each choice set includes five alternatives; the simple number values respectively correspond to the 11 antihyperglycemic medication-specific factors.

**TABLE S2. Subgroup results of CLM**

| **Attributes** | **Male** | **Female** | **From urban area** | **From rural area** | **≥60 years old** | **＜60 years old** |
| --- | --- | --- | --- | --- | --- | --- |
|  | **Coefficient**  **(rank)** | **Coefficient**  **(rank)** | **Coefficient**  **(rank)** | **Coefficient**  **(rank)** | **Coefficient**  **(rank)** | **Coefficient**  **(rank)** |
| Efficacy | 3.146***(1) | 2.640***(1) | 3.629***(1) | 2.296***(1) | 2.789***(1) | 3.011***(1) |
| Cardiovascular health | 2.091***(2) | 1.487***(2) | 2.391***(2) | 1.273***(2) | 1.638***(2) | 1.974***(2) |
| Hypoglycemic events | 1.375***(3) | 0.777***(3) | 1.692***(3) | 0.547***(3) | 0.861***(4) | 1.387***(3) |
| Gastrointestinal side effects | 0.949***(5) | 0.562***(4) | 1.339***(4) | 0.279***(5) | 1.638 (3) | 1.101***(4) |
| UTI and genital infection side effects | 1.119***(4) | 0.416***(5) | 1.202***(5) | 0.344***(4) | 0.541***(5) | 1.072***(5) |
| Edema | 0.521***(6) | -0.232***(8) | 0.619***(6) | -0.316***(7) | -0.121*(7) | 0.525***(6) |
| Out-of-pocket cost (ref.) | -- (9) | -- (6) | -- (9) | -- (6) | -- (6) | -- (9) |
| Weight change | 0.176**(7) | -0.144* (7) | 0.526***(7) | -0.417***(8) | -0.232***(8) | 0.462***(7) |
| Bone fracture | 0.153* (8) | -0.314***(9) | 0.230***(8) | -0.436***(9) | -0.242***(9) | 0.149 (8) |
| Mode of administration | -0.196*(10) | -0.699***(10) | -0.171** (10) | -0.729***(10) | -0.529*** (10) | -0.386*** (10) |
| Dosing frequency | -0.362***(11) | -0.837***(11) | -0.418***(11) | -0.805***(11) | -0.679*** (11) | -0.535*** (11) |

Abbreviations: CLM, conditional logit model；UTI, urinary tract infection；***p *< 0.01*, **p *< 0.05*, **p < 0.1*


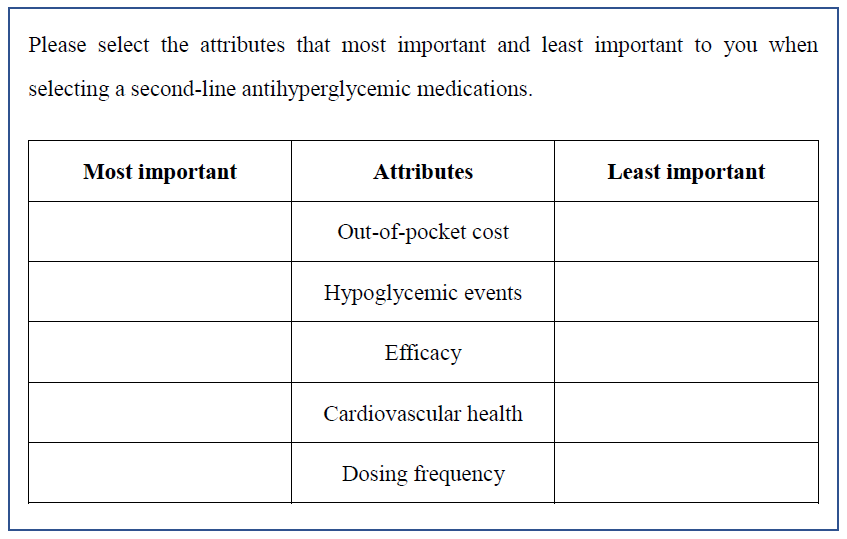


**FIGURE S1.** An example of BWS-1 choice task.


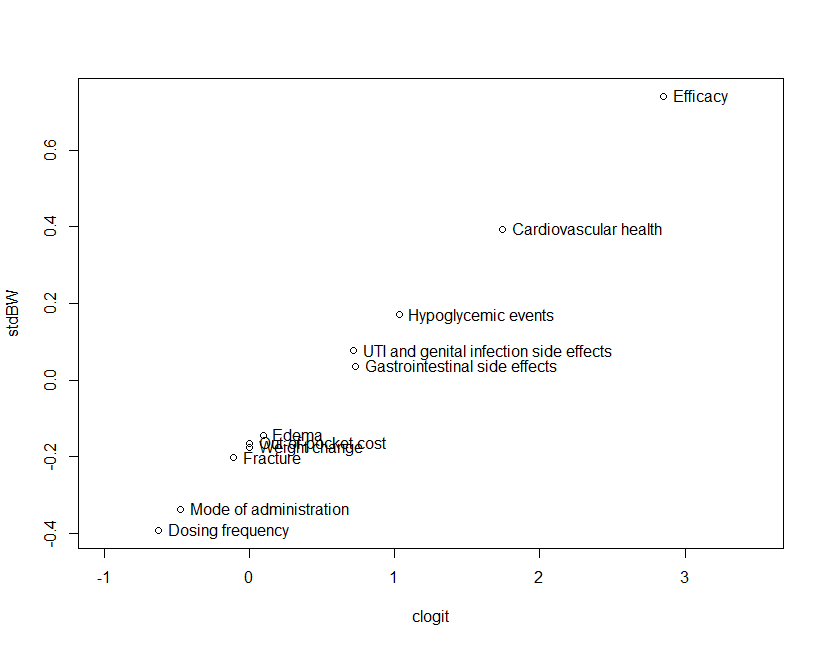


|  | clogit | stdBW |
| --- | --- | --- |
| clogit | 1.000000 | 0.999281 |
| stdBW | 0.999281 | 1.000000 |

**FIGURE S2.** Relationship between the clogit and standardized BW scores.
